# Surgical Management of Latissimus Dorsi and Teres Major Tears in a Water-Skiing Injury: A Case Report and Literature Review

**DOI:** 10.1155/2023/8626419

**Published:** 2023-12-08

**Authors:** Demitri Constantinou, Konstantinos Kastanos

**Affiliations:** ^1^Department of Exercise Science and Sports Medicine, School of Therapeutic Sciences, Faculty of Health Sciences, University of the Witwatersrand, Johannesburg, South Africa; ^2^Linksfield Orthopaedic, Sport and Rehabilitation Centre, Netcare Linksfield Hospital, Johannesburg, South Africa

## Abstract

Tears of the latissimus dorsi and/or teres major tendons are uncommon, with no definitive management. Surgical repair has been reported in high-level athletes, mostly in baseball players. Simultaneous tears of both latissimus dorsi and teres major tendons are rare, with little known of surgical intervention outcomes. We report on the first published case of surgical repair of both latissimus dorsi and teres major tendon tears from a water-skiing injury in a 45-year-old male with favorable outcomes.

## 1. Introduction

The latissimus dorsi (LD) muscle is the largest muscle in the upper body and functions to internally rotate and move the arm inferiorly and posteriorly and extend the shoulder medially, whilst drawing the shoulder downward and backward [1]. The teres major (TM) muscle functions in opposition to the muscles comprising the rotator cuff by extending the arm when it is in the flexed position and rotating it medially [1].

Simultaneous injuries to both the LD and TM tendons are rare. Those that have been reported are primarily in high-level athletes and mostly in professional baseball pitchers [2–4].

There are reports of favorable outcomes in recreational athletes of nonsurgical management, including nonsurgical management in a novice golfer who made good recovery following avulsion of LD and TM tendons. In elite athletes, some authors have suggested primary tendon repair to improve function and retrain to sport sooner [4].

There is a sparsity of data on surgery for injuries of both tendons.

This case report is the first, as far as the authors are aware, of a water-skiing injury with tears of both the latissimus dorsi and teres major tendons and in which surgical repair was performed.

## 2. Case Report

A right-hand dominant 46-year-old male with no significant medical history sustained an injury whilst being pulled out of the water when water-skiing. He felt acute pain in his left shoulder, accompanied by an audible snap. He reported an immediate loss of function in his shoulder. Over the next week, he had an audible click and pain with movement, and although he showed slight improvement in pain, he sought medical attention due to continued dysfunction. Clinical examination revealed a good range of motion of both passive and active flexion (140°), extension (50°), abduction, internal rotation (thumb to T6), and external rotation (50°) with no neurological deficit. There was pain with active shoulder adduction and with internal rotation against resistance and pain inhibition of these affected movements (strength 2/5).

**Figure 1:**
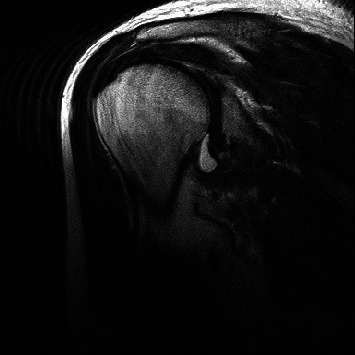
Magnetic resonance imaging coronal view demonstrating disruption and fluid accumulation at insertion of right latissimus dorsi tendon.

**Figure 2:**
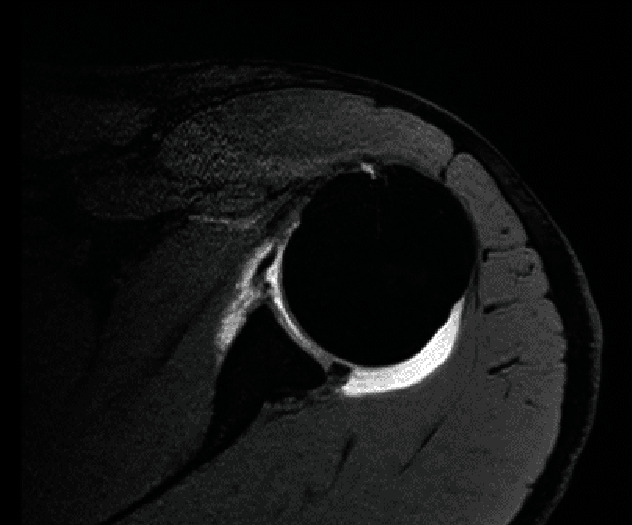
Magnetic resonance imaging axial view demonstrating disruption of right latissimus dorsi tendon.

An MRI scan with pre- and postgadolinium arthrogram was performed. This suggested a type II SLAP lesion and complete tear of the latissimus dorsi tendon insertion onto the humerus (Figures 1 and 2). There was no associated bone avulsion visible. The teres major tendon injury was not obvious on the scan as a separate injury, even with retrospective review, perhaps due to the tendon injury being at the junction of the larger LD tendon.

Due to the rarity of the injury, there is no established superlative treatment approach; the treatment options were discussed with the patient. Both surgical management and nonsurgical management were considered including rehabilitation, levels of activity, risks and benefits, and possible outcomes. In relation to surgical repair, neurological harm and other potential surgical complications were detailed to the patient. Based on this, the patient made the informed choice to have surgical intervention with primary repair of the torn tendon.

**Figure 3:**
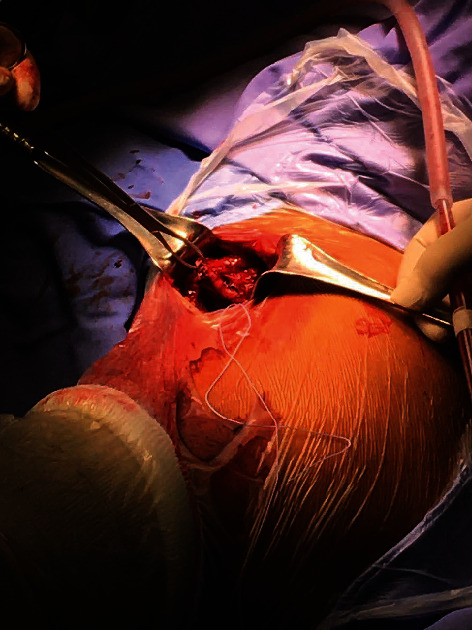
Surgical exposure revealing tears in latissimus dorsi and teres major tendons. The top of the picture is proximal, the bottom left is distal arm (covered by stockinette), and the right-hand side is medial.

**Figure 4:**
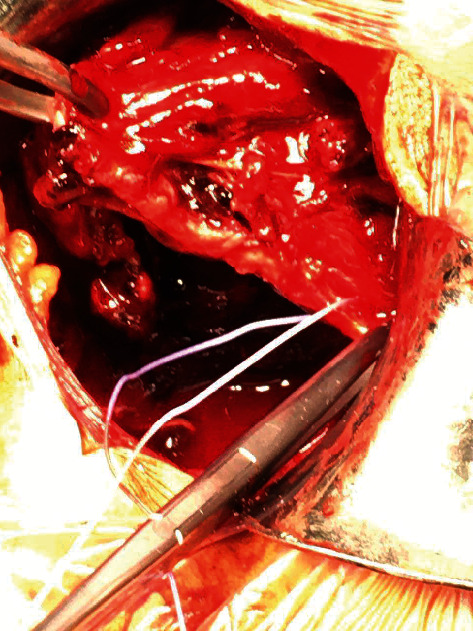
At surgery, tears of both latissimus dorsi and teres major were identified. The top of the picture is the proximal arm, the bottom is distal, and the right-hand side is medial.

At surgery (Figures 3 and 4), in the right decubitus position, a posterior axillary fold chevron incision was made. The LD was identified, and lack of tension in the tendon was noted. The tendon was followed proximally. Bruising and oedema were noted. To allow retraction of the triceps and teres minor tendons, the shoulder was abducted and internally rotated. A contained haematoma was encountered, which was evacuated. Complete rupture of both the LD and TM tendons were recognised. The tendon margins were debrided. The bare insertion at the humerus was readily palpable and visible and was debrided. Repair of the combined tendon mass was performed with ORTHOCORD™ (DePuy Mitek, Massachusetts, United States) Mason-Allen sutures and Arthrex™ Pec Buttons (Munich, Germany). Three of these constructs were inserted. The repair was readily achievable. The surgical site was thoroughly irrigated out and haemostasis confirmed. Layered closure was performed using absorbable sutures. A padded dressing was applied and shoulder immobilisation achieved with a sling.

**Figure 5:**
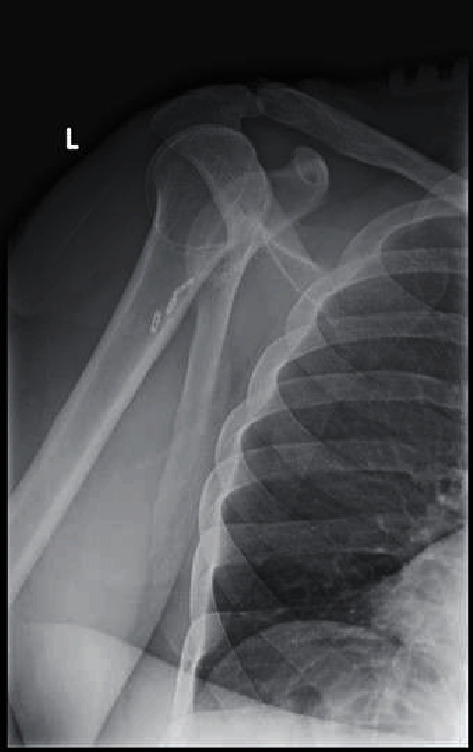
Postoperative radiography demonstrating anchor placements.

Postoperative transcapular radiological views revealed the in situ anchor devices and mild posterior subluxation of the humerus head (Figure 5).

At 4-week postoperative follow-up consultation, the patient did not report any problems. He confirmed compliance with using the sling. The skin wound was clean, and there were no neurological injury signs. He was advised to continue with light activities of daily living and referred for physiotherapy, targeting scapula movement and range of motion. This included warm-up, active range of motion (ROM) of upper limb, passive ROM in forward flexion, elevation in scapula plane, and external rotation. Rehabilitation progressed from week 6 with passive ROM in forward elevation to 90° and external rotation to 30°.

At 5 months after initial physiotherapy, the patient had undergone final phase rehabilitation with strengthening of deltoid, periscapular muscles, internal and external rotation, biceps, and triceps all in supine and lying position and progressed to standing/seated positions.

He was satisfied with his functional progress. The latissimus dorsi and teres major mass was palpably active. Active shoulder elevation reached 140°, external rotation to 50°, and internal rotation thumb to T8. There was no clinical weakness. He was referred progressive return to sport rehabilitation with progression to return to swimming.

At 32 months, he reported being pleased with the outcome of the procedure. Activities of daily living had normalised. The patient had returned to exercising in the gym without any difficulty, except for pull-ups, which he self-restricted. He had successfully returned to open water swimming (1-mile event). Examination revealed symmetrical posterior axillary fold contour and bulk. Left scapula stability was paradoxically better than the right, probably due to asymmetrical rehabilitation.

## 3. Discussion

Water-skiing injuries are primarily reported as being traumatic and include falls, propeller-related incidents, and injuries of the lower limb [5]. Most reported tendon injuries are of the hamstring muscle [6].

Erickson et al. [3] reported on 11 baseball players that underwent surgical repair of latissimus dorsi and/or teres major. They did not, however, report how many of those had injuries of both the latissimus dorsi and teres major tendons. They did report that all 11 returned to their athletic activities postsurgery. Donohue et al. [2], in a review, reported 55 injuries of LT and/or TM tendons with 32 in baseball. Only 3 were of both tendons (2 in baseball and 1 in golf). They described that 5 cases were injuries from water-skiing, of which 3 were of the latissimus dorsi tendon and 2 of the teres major tendon. None was both latissimus dorsi and teres major tendon tears. In that report, two of the latissimus dorsi water-skiing injuries were surgically repaired. They further reported in their review that “all patients eventually return to play with full range of motion and full subjective strength.”

In light of our case, in those patients of isolated LT or TM tears that were treated nonsurgically, one needs to consider injuries to both tendons that have been missed clinically and on MRI scans.

Surgical management and nonsurgical management have been reported [2], with nonsurgical management sufficing for latissimus dorsi injuries, particularly for recreational athletes [2, 3]. The only reported cases of isolated teres major tears in professional ice hockey players were treated nonsurgically with rapid return to activity. There are no randomized studies to compare outcomes of surgical versus nonsurgical management. Other reports of surgical repair are with chronic latissimus dorsi tear-related degenerative pathology and not traumatic.

The latissimus dorsi and teres major musculotendinous complex contributes to complex shoulder functions; particularly, they together extend the arm and rotate it internally. Although rare, injuries to either of these structures have been reported, more so of the latissimus dorsi. These occur mostly in baseball players with few occurrences of both tendon ruptures occurring simultaneously.

Injuries reported include a partial tear of latissimus dorsi in water-skiing and two cases (servicemen) with latissimus dorsi tendon avulsion, which were managed nonoperatively.

Surgical repair in a water-skier for acute latissimus dorsi avulsion has been reported [7]. Surgical repair for a complete LD tendon tear in a semiprofessional rock climber was reported with good outcomes and return to rock climbing at 9 months with continued improvement at 16 months [8].

A 38-year-old male competitive slalom water-skier had a LD tendon tear repaired with suture anchors, with postsurgery isokinetic testing showing total work performance not only restored but better in comparison to the nonoperated shoulder. Our patient likewise exhibited clinical stability better than the uninjured side at 32-month follow-up. This is likely due to the compliance and concerted effort of rehabilitation of the injury.

The first water-skiing injury with the uncommon isolated tear of TM was reported by Maldjian et al. [9]. There have been reports of nonoperative management of isolated TM tendon tear in water-skiers. The mechanism of these injuries was all related to the arms being pulled forward as the boat accelerated. This was in keeping with the reported mechanism of injury in our patient.

However, to date, no combined traumatic LD and TM tendon tears have been reported in water-skiing and undergoing surgical repair.

In our case, an injury whilst water-skiing presented clinically with dysfunction and signs suggestive of LD rupture. The injury occurred when being extracted from the water, a process known to exert substantial forces (equivalent to one and a half times the body weight) on the upper extremities [10]. Takeoff forces transmitted to the upper extremity during water-skiing (Orthopedics, 26 (7), 707-710).

Maldjian et al. [9] postulated the anatomic and kinematic factors of shoulder extensor (teres major and latissimus dorsi) injuries in water-skiing. To resist the force of the held rope tightening as the boat accelerates, these extensors oppose flexion of the upper limb. The teres major is at greater risk, due to its insertion further away from the axis of rotation and inferior to the rotator cuff, where a greater force acts on it, and in a stretched position, when the arm is flexed. The rotator cuff exhibits less constraint in that position. They further postulated that although the insertion of latissimus dorsi is anatomically close to that of teres major, it is less at risk due to its larger size. This suggests that in our patient, significant forces were at play to result in tears of both tendons. The suspected latissimus dorsi tear was confirmed by MRI, but at surgery grade 3, tears of both latissimus dorsi and teres major tendons were evident. Of 5 cases in the literature of similar injuries, caused by activities other than water-skiing, 2 were treated surgically. It is known that some tendon injuries require surgery to restore function and include quadriceps tendon (common in over 40 year olds), patellar tendon, peroneal tendon, Achilles tendon, and hip abductors. Surgical outcomes are positive with repairs of rotator cuff, pectoralis major tendons, and subscapularis. Outcomes for tendon repairs vary depending on whether they are traumatic or degenerative, and there may be age-dependent variables. It has been shown that shoulder tendon repairs in under 40 year olds performed arthroscopically recover adequate function.

Our patient was over 40 years old at the time of injury and, after discussion of options, opted for surgery. The surgical procedure was technically without complication; the patient was compliant and underwent graded return to activities whilst undergoing rehabilitation. The immediate- to intermediate-term postoperative course was uneventful. As improvement may be expected beyond 16 months [8], our patient was followed up to 32 months.

The limitations of this case include that it is just one case study, and outcomes of other interventions or management are unknown.

## 4. Conclusion

Surgical repair of combined latissimus dorsi and teres major tears in a 45-year-old with a water-skiing accident was technically feasible, with good short- and medium-term clinical and functional outcomes. Future possible studies are required including need of further comparison of surgical vs. nonsurgical outcomes of these injuries in elite athletes as well as in the general population.

## Data Availability

The data used to support the findings of this study are restricted by the Wits Human Research Ethics Committee (Medical) in order to protect patient privacy. Data are available from Demitri Constantinou (demitri.constantinou@wits.ac.za) for researchers who meet the criteria for access to confidential data.
